# Investigation of Novel Solid Dielectric Material for Transformer Windings

**DOI:** 10.3390/polym15244671

**Published:** 2023-12-11

**Authors:** Aysel Ersoy, Fatih Atalar, Alper Aydoğan

**Affiliations:** 1 Department of Electrical and Electronic Engineering, Istanbul University-Cerrahpasa, Bağlariçi St. No. 7, Avcılar, 34320 Istanbul, Turkey; fatih.atalar@iuc.edu.tr; 2 Computing & Information Services Office, Muğla Sıtkı Koçman University, Kötekli Campus, 48000 Muğla, Turkey; alperaydogan@mu.edu.tr

**Keywords:** pressboard, polyurethane, harmonics, transformers mineral oil, breakdown

## Abstract

Improvement techniques aimed at enhancing the dielectric strength and minimizing the dielectric loss of insulation materials have piqued the interest of many researchers. It is worth noting that the electrical breakdown traits of insulation material are determined by their electrochemical and mechanical performance. Possible good mechanical, electrical, and chemical properties of new materials are considered during the generation process. Thermoplastic polyurethane (TPU) is often used as a high-voltage insulator due to its favorable mechanical properties, high insulation resistance, lightweight qualities, recovery, large actuation strain, and cost-effectiveness. The elastomer structure of thermoplastic polyurethane (TPU) enables its application in a broad range of high-voltage (HV) insulation systems. This study aims to evaluate the feasibility of using TPU on transformer windings as a solid insulator instead of pressboards. The investigation conducted through experiments sheds light on the potential of TPU in expanding the range of insulating materials for HV transformers. Transformers play a crucial role in HV systems, hence the selection of suitable materials like cellulose and polyurethane is of utmost importance. This study involved the preparation of an experimental setup in the laboratory. Breakdown tests were conducted by generating a non-uniform electric field using a needle–plane electrode configuration in a test chamber filled with mineral oil. Various voltages ranging from 14.4 kV to 25.2 kV were applied to induce electric field stress with a step rise of 3.6 kV. The partial discharges and peak numbers were measured based on the predetermined threshold values. The study investigated and compared the behaviors of two solid insulating materials under differing non-electric field stress conditions. Harmonic component analysis was utilized to observe the differences between the two materials. Notably, at 21.6 kV and 25.2 kV, polyurethane demonstrated superior performance compared to pressboard with regards to the threshold value of leakage current.

## 1. Introduction

The maintenance of dielectric strengths for solid insulators used in transformers is crucial in ensuring the continuity of energy supply. Solid insulators operate in the same environment as mineral oil, a petroleum-based liquid dielectric, but may experience a decrease in insulation performance over time [1,2]. Despite periodic maintenance being of great importance, the deterioration of solid insulators is considered an irreversible condition [3]. Hence, ensuring healthy operation of the transformer mainly relies on appropriate solid insulator selection and testing. Pressboard materials based on cellulose are typically used as solid insulators [4,5] in oil-type transformers [6]. Nevertheless, transformer stability can be achieved by utilizing insulators with high dielectric strength like polyurethane. Therefore, it is advisable to consider conducting appropriate tests on polyurethane materials to enhance insulation resistance.

In their studies, Aruna et al. investigated several options for paper insulators. They compared the electrical properties of cellulose film with those of bacterial cellulose and also examined their advantages, disadvantages, and nuclear magnetic resonances. Moreover, they found that electrical loading, operating temperatures, and water level in oil have a significant impact on the deterioration of cellulose insulation [7]. The study analyzed the heat behavior of different papers in oil-impregnated paper insulators under heat-induced aging. Morooka et al. determined that kraft paper’s flexibility decreases over time and its modulus of elasticity reduces, which jeopardizes the insulation system [8].

The insulation characteristic of a transformer can be evaluated using impulse, direct current (DC) or alternative current (AC) voltages. Nedjar carried out a reliability analysis of paper insulators using both AC and DC voltages by applying step voltage. The results showed that discharges occur when the electric field applied to the pressboards exceeds the critical value, and these discharges occur between oil and pressboard. A conductive path is formed in the process, which has been observed to degrade the insulation system. It has been reported that the resistance of oil-impregnated pressboards to DC voltage is superior to that of AC voltage, according to a reliability analysis based on step voltage practices [9]. The modelling of lightning strikes in transformers was undertaken by Florkowski and et al. They discovered that the strength of the windings is influenced by the waveform of the applied voltage and the standard voltage pulse used. It has been established that overvoltage mainly impacts the insulation system windings and ground electrode. According to reports, overvoltage has reached its maximum value. The insulator has been found to be adversely affected by this situation at standard impulse voltage. However, there were no observed effects on the insulator interface at discrete impulse voltages [10].

The investigation into the exterior design of transformers is ongoing. Chaw et al. conducted a comparison between the rectangular and circular winding distribution transformers at the 1000 kVA level. Consequently, models were created for 1000 kVA 50 Hz 11/0.4 kV three-phase, two-winding, triangular star-connected core distribution transformers. It is predicted that efficiency will improve as transformer losses decrease. The magnetic voltage is anticipated to differ between the proposed transformers and the target design, as the losses in the transformers are reduced [11].

It is crucial to consider the variability of experimental methods when assessing the condition of a transformer, along with the significance of the recommended oils and insulation materials used in transformer insulation. The condition of the oil plays a critical role, as demonstrated in research conducted by Malik Yadav, Mishra, and Mehto, investigating transformer breakdown and maintenance scenarios. The study compared traditional methods against the neuro-fuzzy technique [12].

Pradhan and Yew conducted a frequency field analysis of data obtained from their experimental study and evaluated it using harmonic values [13]. Sun et al. examined the efficacy of fiber coating materials in power transformers and demonstrated the superiority of ethylene tetrafluoroethylene (ETFE), polytetrafluoroethylene (PTFE), and polyamide 12 (PA 12) over greaseproof paper [14]. Substitutions in electromagnetic field distribution, particularly in windings, are currently intensively studied in high voltage transformers and distribution transformers. Sun et al.’s modelling study indicated that the increase in pressboard thickness from 5 mm to 10 mm could result in a change of up to 30% in electric field decrease [15].

This study compares the effectiveness of pressboard, a cellulose derivative, and polyurethane, a common material in the electrical industry, as a transformer insulator. We measured the peaks of leakage currents flowing from the earth electrode after applying voltage to the prepared insulating plates, and we calculated the harmonics as well. In our study, we have experimentally researched and provided data on the breakdown behavior when using polyurethane and polymers as alternatives to paper insulators. To accurately analyze surface catastrophic events, we used scanning electron microscopy (SEM) images of each case and identified the superior cases of TPU.

## 2. Measurement Methodology

The study measured leakage currents on the earth electrode using the Fluke ScopeMeter 199C series oscilloscope, which belongs to the Fluke brand and is rated as CAT IV Class. These oscilloscopes have a sampling rate of 2.5 GS/s (giga samples/second) and a bandwidth of 200 MHz. Measurements were recorded graphically and evaluated for a 6 s duration, with a total of 30,000 data points. The FlukeView^®^ SW160 software, with a multi-function counter interface, was purchased and used to process the data in MATLAB 2022b software.

**Figure 1:**
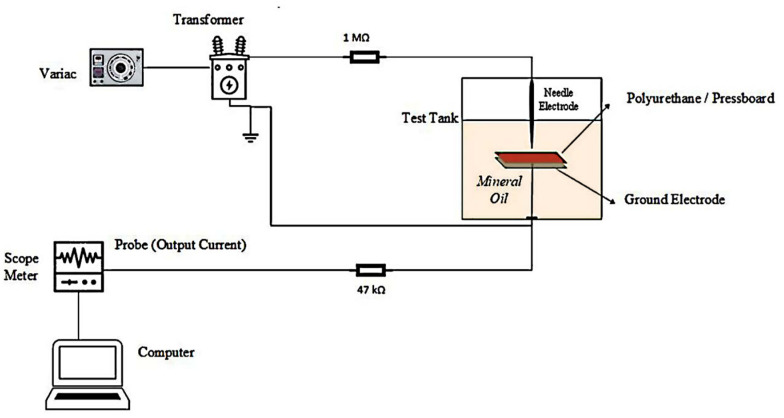
Electrode system for surface electric discharges, with solid dielectric made of pressboard and polyurethane.

Various assessment techniques and norms are available to evaluate the partition between solid and liquid media within transformers as well as to comprehend the partial discharge that may arise. Specific tests have to be executed, predominantly in the case of cellulose-based pressboards. A test mechanism was established in this investigation, making use of pertinent standards as a basis [16,17]. The pressboard is of 100 × 100 mm dimension and it has fiber density of 1.2 g/cm^3^, a relative dielectric constant of ε_r_ = 4.1 and the loss factor is 0.0057. The relative dielectric constant of mineral oil is ε_r_ = 2.2. Also, the PU has same dimension of pressboard. Its relative dielectric constant of ε_r_ = 12.25 and the loss factor is 0.0021. The investigation involved the initial recording of leakage current signals. Disturbance points in the leakage current signal waveform are attributed to partial discharge. The study analyzed and interpreted the peak values at these points. The testing equipment and measurement system used in this study are identical to those employed in our earlier research [18]. The main objective of the present study is to compare two dissimilar types of solid insulating materials. The electrode system setup, used for testing the proposed PU and pressboard, is displayed in Figure 1. The thicknesses of the dielectric materials examined in the investigation are 1 mm, 2 mm, and 3 mm. In addition, the mineral oil level remained unvaried throughout the research. The voltage levels were varied, though. Four voltage levels ranging from 14.4 kV to 25.2 kV were applied to the insulators. Accordingly, the leakage current threshold values during partial discharge were determined. The peaks of the measured values that exceeded this threshold value for the applied voltage level indicate the commencement of partial discharge and result in signal deterioration [19,20,21,22].

## 3. Results and Discussion

### 3.1. Leakage Current and Harmonic Analysis

The number of current peaks and values were calculated at all voltage levels for the pressboard of 3 different thicknesses. Values for these data are given in Table 1, Table 2 and Table 3, respectively, for 1 mm, 2 mm, and 3 mm thicknesses.

Threshold values of flowing currents are selected with similar values for each solid insulator. As can be seen in Table 1, pressboard has more peak points even though its threshold is slightly less than polyurethane at the applied lowest voltage level. When it comes to the situation at the highest voltage level, polyurethane shows worse insulation performance. It can be concluded from this point that pressboard can be selected for higher voltage levels at the smallest electrode gap. However, polyurethane has more strength when the voltage level is 14.4 kV. So, new investigations are needed to clarify interpretation with new electrode gaps. Table 2 consists of the data of two solid insulators at the thickness of 2 mm.

As shown in Table 2, there is no significant difference between two solid insulators at the voltage level of 14.4 kV and 18 kV. PU has higher peak current value at 21.6 kV. At this voltage level, PU is more advantageous to select as an insulator. However, at the highest voltage level, even though pressboard has higher peak current value, its peak points are less than PU. So, we have to select a new electrode gap to make more reasonable comparison for selecting between PU and pressboard. Table 3 shows the same manner of data of PU and pressboard at the thickness of 3 mm.

As can be seen from Table 3, there is no significant difference between the two insulators at the 14.4 kV voltage level. At this level, only at the point where the current signal is disturbed, the peak number of signs with partial discharge is higher in the pressboard. This makes the polyurethane material a step above the pressboard. When the 18 kV and 21.6 kV voltage levels are examined, it is seen that the pressboard leakage current is 40 mA more than that of the polyurethane. There are also serious differences between the discharge peak points for these levels. At 25.2 kV voltage level, there is 80 mA difference between the leakage currents measured from PU and pressboard.

The peak values and number of these points of leakage current are not enough to make a more comprehensive evaluation in terms of the dielectric strength behavior of PU and pressboard. So, in this study, harmonic current values between 50–1000 Hz frequency band are calculated for every special situation. Another purpose of harmonic current performance is to determine the dominant harmonic component of current signal. With the help of this method, there is an opportunity to make a clearer evaluation in dielectric behavior differences. The harmonic current values of PU and pressboard at the 1 mm electrode gap are summarized in Table 4.

There is an interesting common situation for both solid insulators in terms of dominant harmonic values. It can be clearly seen in Table 4 that 2nd and 6th harmonic values have higher values than the others at the all-voltage levels for PU and pressboard. These values were calculated very close to each other for both materials. Therefore, it can be said that the leakage currents for 1 mm thickness are similar for both insulators. In this case, it is necessary to examine the harmonic values for 2 mm thick materials, as given in Table 5.

From Table 5, it is seen that there is similar behavior in terms of dominant harmonic values with 1 mm material thickness. So, it can be said that the electric field creates similar stresses on the materials of both 1 mm and 2 mm thickness. However, at the voltage levels of 21.6 kV and 25.2 kV, the fundamental components (50 Hz) values of PU are higher than the counterpart of pressboard. It can be said that harmonics, except from the fundamental component, are seen more intensely on pressboard. So, when the 2 mm thickness material is used at higher voltage levels, the PU selection is more reasonable than pressboard. This situation is supported by higher peak current value of pressboard at the highest voltage level, as seen in the Table 2 data.

Harmonic values at the 3 mm thickness are summarized in Table 6. At the voltage level of 14.4 kV, the 3rd and 7th harmonics were measured as 23 mA and 10.93 mA for pressboard. No double harmonics have been observed in the leakage current components. The number of peaks exceeding the peak value threshold is higher in the pressboard than in polyurethane. While examining harmonics for 18 kV voltage level, 3rd harmonics are measured as 3.1 mA for both pressboard and PU. The calculated 5th harmonic component is 17.9 mA for pressboard and 15.7 mA for polyurethane; pressboard’s 5th harmonics are higher than polyurethane. The 13th harmonic value is similarly higher on the pressboard; this level is 9 mA for pressboard and 3.6 mA for polyurethane. The leakage current values for the 15th and 17th harmonics were measured as 5 mA and 2.8 mA, respectively, for the polyurethane, and 8.9 mA and 5.6 mA, respectively, for the pressboard. At these levels, the 15th and 17th harmonic components are higher than PU.

No comparable 3rd harmonic component was observed for the voltage level of 21.6 kV. The 5th harmonic value was measured as 21.8 mA on the pressboard and 21.4 mA on the polyurethane. This apparent difference offers the possibility of evaluation and comparison in terms of partial discharge.

**Figure 2:**
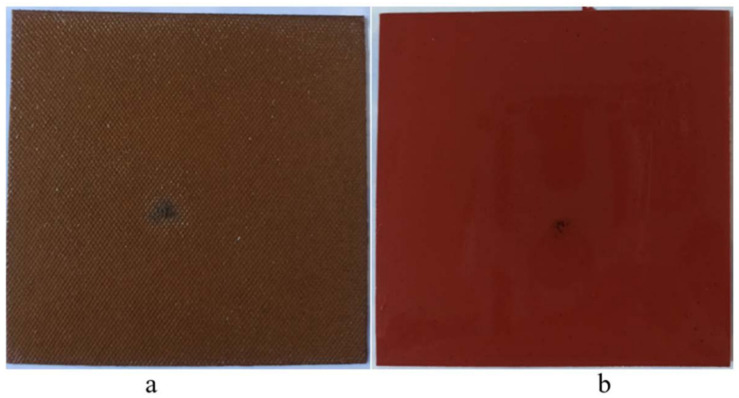
Surface breakdown points at 25.2 kV ((**a**) pressboard and (**b**) polyurethane).

The surface condition of insulators is shown in Figure 2 at the thickness of 3 mm. It can be clearly seen that the diameter of the surface carbonization pattern of pressboard is wider than the polyurethane. This situation can be a determinant of the dielectric strength of these solid insulators in the oil. PU has more durability against the formation of surface tracking formation. There is no comparable surface condition for 1 mm and 2 mm material thickness. It will be given a detailed surface morphology examination in the following SEM section.

### 3.2. Scanning Electron Microscopy (SEM) Analysis

Initially, we applied breakdown stresses on materials with similar thickness in the experimental setup and compared the outcomes. Subsequently, we analyzed and interpreted size variations of the same materials along with the reactions and behavior patterns of different materials with similar sizes through SEM photos of the resultant material.

**Figure 3:**
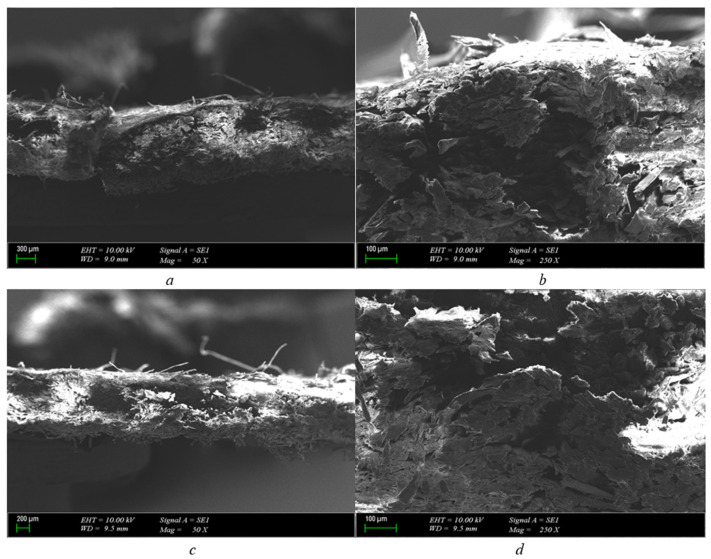
1 mm pressboard breakdown in 25.2 kV SEM photos (scales are (**a**) = 300 μm-50×, (**b**) = 100 μm-250×, (**c**) = 200 μm-50×, and (**d**) = 100 μm-250×).

When examining the 1 mm pressboard material with a 1 mm electrode gap, as Figure 3a illustrates, we see a drilled hole running through the center of the pressboard, which constitutes a compressed paper structure. The point of breakdown indicates darkening, though no layered structure distortion was noted. Furthermore, it was observed that a 400-micron hole had been drilled in the pressboard’s interior. The burn does not progress through infiltration, as evident from these findings. The carbonized path was anticipated to move inward, but it has yet to materialize, as evidenced by its current location. The expected structure has not been formed as a result. The experiment was promptly terminated after the breakdown occurred.

As demonstrated in Figure 3b, a 400-micron hole was created in the 1 mm pressboard insulator following a breakdown at the specified voltage. The carbonized path did not propagate throughout the pressboard, leading to immediate termination of the experiment upon breakdown. In addition, a weak spot in the pressboard caused an explosion after the breakdown test. The dielectric behavior observed did not disrupt other layers since the pressboard (Figure 3c), which possesses a cellulosic structure, lacks deceleration between layers due to its fibrous structure. Consequently, no significant structural changes occurred.

The cross-section obtained through the pressboard reveals explosion-shaped zones indicating micro-level physical deformation formed on the paper after electrical breakdown. However, the deformation did not exceed the layers found in the fibrous structure of the pressboard, and, as such, no significant structural changes occurred.

**Figure 4:**
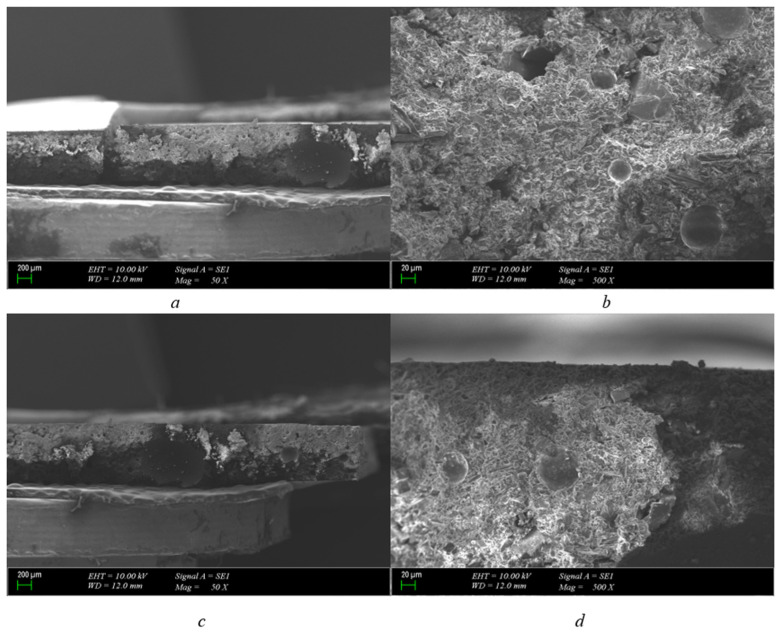
1 mm polyurethane breakdown in 25.2 kV SEM photo (scales are (**a**) = 200 μm-50×, (**b**) = 20 μm-500×, (**c**) = 200 μm-50×, and (**d**) = 20 μm-500×).

It is clearly seen in Figure 4a that polyurethane is a structurally hollow material. The hollow state of the structure is remarkable in the tests carried out with polyurethane samples, which are also used as insulators in cable headers commercially. It is obvious that it will show an effective behavior in the face of volumetric stresses in polyurethane material just like in the same thickness pressboard. 

Pressure is formed on the pressboard, which is located inside the oil. It is not easy to see that the transformer has bombed due to this pressure. This is due to the fact that the paper absorbs pressure. In the same case, it is also seen that, due to the convex structure found in polyurethane, it works as actively as the pressboard in absorbing this pressure and its availability instead of the pressboard is observed.

When the polyurethane material was approached and examined with a 20 μm scale, the carbonized region of the structure in the polyurethane material was not disturbed. Just like in the pressboard material, it seems that the breakdown ends with a regional burn.

There are no gaps or traces of carbon along the channel. The breakdown is limited to this area, as in the case of a 1 mm pressboard, so, in the case of a 1 mm polyurethane insulator, the material is structurally very thin. Therefore, if we interpret the breakdown, it is seen both more quickly and as a point distortion. If the structure of the material was thicker, the possibility that the breakdown would progress and form a carbonized path could have been considered, but this did not happen for a 1 mm structure.

The 200 μm scaled cross-section shows that the breakdown occurred at a single point, and the convex structure of the polyurethane material absorbed the resulting pressure, preventing the sudden expansion and progression of the breakdown. As can be seen in Figure 4d, when 20 μm was approached to the polyurethane from a different cross-section, it was seen that the progress after the breakdown did not lead to a carbonization.

**Figure 5:**
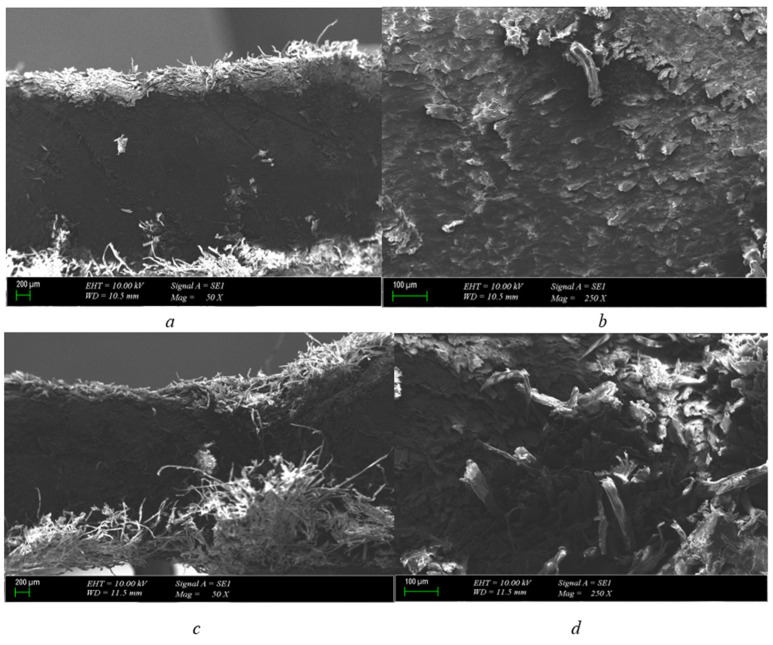
2 mm pressboard breakdown in 25.2 kV SEM photo (scales are (**a**) = 200 μm-50×, (**b**) = 100 μm-250×, (**c**) = 200 μm-50×, and (**d**) = 100 μm-250×).

As can be seen from Figure 5a, the inner part of the 2 mm pressboard structure is chipped. After the breakdown, a burn was formed. As a result of combustion, a duct was formed. There is progress along this channel; why did combustion occur along a channel for a 2 mm material when there is only a point discharge for a 1 mm pressboard material? The answer to this question is as follows. This material will not break down immediately and will not tear. As can be seen from Figure 5b, the structure is carbonized from the traces formed on the 2 mm pressboard compared to the 1 mm pressboard.

In Figure 5c, when a different cross-section of the pressboard formed after the breakdown voltage is examined, the fibrous structure of the paper formation and cellulosic material is clearly seen. The combustion caused by the breakdown inside the pressboard and the carbonized path formed at the end of it have been clearly revealed. As can be seen in (Figure 5d), the burns that occur in the cellulosic structure are soon clearly visible. The deterioration of the structure as a result of carbonization gives us an observation idea about the resistance of the material to breakdown.

**Figure 6:**
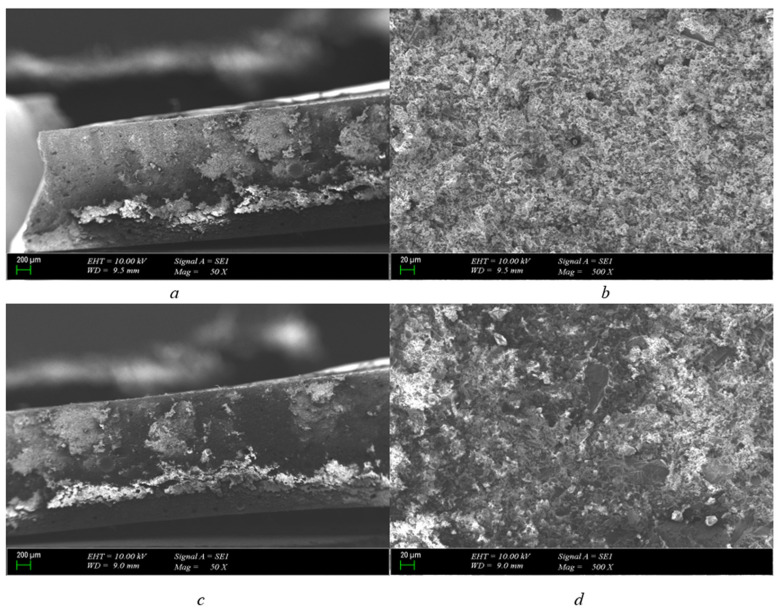
2 mm polyurethane breakdown in 25.2 kV SEM photo (scales are (**a**) = 200 μm-50×, (**b**) = 20 μm-500×, (**c**) = 200 μm-50×, and (**d**) = 20 μm-500×).

2 mm polyurethane 200 μm cross-section is presented in Figure 6a. A similar progression that occurs on a 2 mm pressboard is also observed on polyurethane. As a result of the breakdown, the burn formed a carbonized path, but its difference from the pressboard is clearly visible. Structurally, there is a wider distortion of the pressboard. In polyurethane, the channel is much thinner. When 2 mm pressboard and polyurethane material are compared, it is seen that the structural deterioration of cellulosic paper is worse than polyurethane.

After the applied voltage is terminated, it is seen that polyurethane is structurally more robust when we enlarge it according to the 20 μm scale to examine the structure. Unlike the pressboard, it seems that polyurethane with the same mm scale does not have a hole channel as large as the one on the paper. In addition, there are no structural breakdowns. Structural bubbles contained in polyurethane help to preserve their structure due to the fact that they are trapped under pressure and become old again in a free state. When the scale is reduced (Figure 6c), the burns and channels on the polyurethane are more clearly visible. But it seems that it is not as large as the channels located on the pressboard of the same thickness. Despite this, the carbonized structure formed by burns is more clearly present here.

When 2 mm polyurethane taken from a different cross-section of the material is examined (Figure 6d), it is seen that the structural integrity is maintained and it is better than the pressboard. But, at the same time, the carbonized breakdown formed as a result of combustion can be seen in this section.

**Figure 7:**
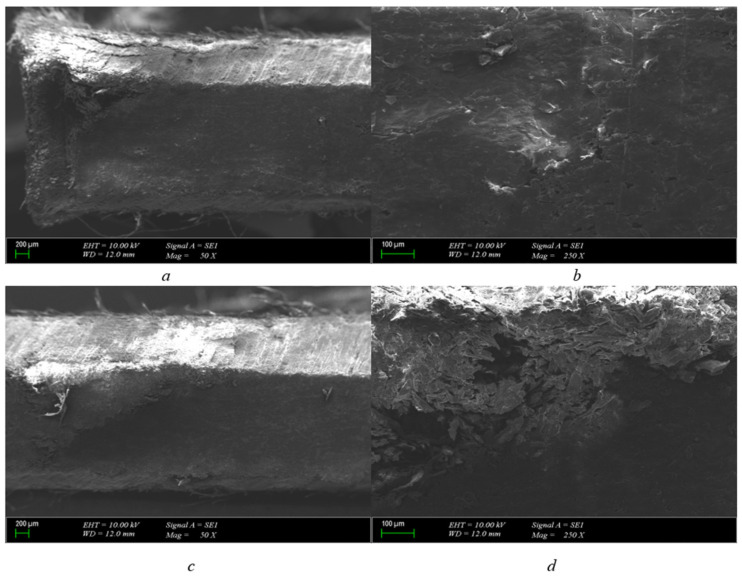
3 mm pressboard breakdown in 25.2 kV SEM photo (scales are (**a**) = 200 μm-50×, (**b**) = 100 μm-250×, (**c**) = 200 μm-50×, and (**d**) = 100 μm-250×).

As can be seen from Figure 7a, there is no breakdown on the outside of the pressboard, but there is burning on the inside. Disturbances have occurred in the internal structure. Due to the increase in thickness of the pressboard, partial discharges are observed on the inside.

In (Figure 7b), it is seen that combustion occurs when the cross-sectional scale for the pressboard is enlarged, but the cellulosic structure is not as deteriorated as in the 1 mm and 2 mm pressboards. The fibers of the paper structure are most degraded on the 1 mm pressboard; for a 2 mm pressboard, less distortion is visible. The conclusion that can be drawn from this is that as the thickness of the pressboard is increased, the cellulosic fiber structure is less impaired in breakdown resistance.

It can be said that although the breakdown is not observed from the outside in the section examined for the 3 mm pressboard (Figure 7c), holes are formed as a result of burning on the inside, and, as a result, a breakdown may be present from the inside out. It has been found that while there is no breakdown outside the cellulosic fibrous structure on the pressboard, the high voltage that causes the breakdown inside can penetrate through the hole in the smallest cellulosic structure and cause explosions inside.

When the cross-section of the area (Figure 7d) is examined, it is seen that combustion disrupts the cellulosic structure inside. It has been determined that the reason for this formation is that the breakdown tension does not proceed from a single point by damaging the fibrous structure, but creates combustion at multiple points independent of each other.

**Figure 8:**
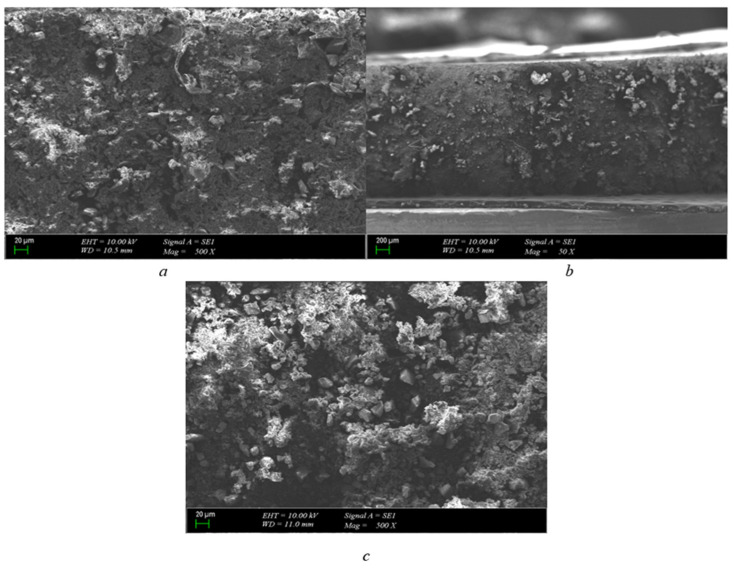
3 mm polyurethane breakdown in 25.2 kV SEM photo (scales are (**a**) = 20 μm-500×, (**b**) = 200 μm-50×, and (**c**) = 20 μm-500×).

As can be seen when the polyurethane material is approached by scaling 20 μm in Figure 8a, large holes formed as a result of combustion are not visible as in 2 mm polyurethane, as the material thickness increases. But it can be said that fragmentary small discharges are formed and are independent of each other, and the breakdown voltage causes more than one distortion in the weak places of the material.

When the size of the scale is reduced to 200 μm (Figure 8b), there is an opportunity to study the structure of the material from a broader point of view. At the end of this, we see that the structure remains more stable overall. It can be said that the bubbles of polyurethane are permanently damaged by breakdown, but large holes are not formed.

The SEM photo of 3 mm polyurethane is shown in (Figure 8c). This is better to see that the structure’s domes are broken here. It is expected that the dome structure will better repel the pressure created by the breakdown voltage created as the material thickness increases and protects the dome structure. But, as can be seen from the figure, the convex structure is best in 1 mm polyurethane, while 2 mm is slightly more distorted in polyurethane; on the other hand, the most distorted state has appeared in 3 mm polyurethane.

## 4. Conclusions

The effective dielectric performance of the solid insulator in mineral oil is critical for ensuring transformers operate safely and without interruption. A high level of insulation resistance is necessary to prevent any potential short circuits and protect the transformer windings. This study investigates the electrical properties of PU and pressboard dielectric materials at thicknesses of 1 mm, 2 mm, and 3 mm. The experiments were conducted using a needle–plane electrode configuration in oil, under a non-uniform electric field. The harmonic values, peak points, and current values were measured at material thicknesses of 1 mm and 2 mm, but no clear selection criteria were identified for choosing between PU and pressboard. Even though these thicknesses lack precise interpretation, their consideration within the philosophy of science marks a new contribution to the literature. At a thickness of 3 mm, it can be stated that polyurethane (PU) offers superior insulation resistance in comparison to pressboard. As the thickness of the material increases, the dielectric performance of pressboard decreased. Although cellulose structure provides an advantage in the absorption of transformer oil, it can be said that the discharges occurring within the structure rapidly propagate and separate into channels. In this case, the leakage current flows faster in these channels and reduces the strength of the material. For thicker samples, the area in which electrons can move and form channels increases. The experimental study shows that this situation leads to more destructive effects on the pressboard. Experimental studies have shown that pressboard exhibits higher harmonic components at the same voltage levels for a 3 mm thickness. It is currently not possible to conclude that the use of polyurethane is definitively better than pressboard. Nevertheless, it is important to consider the interpretation of harmonics in the calculation of material lifespan. Although pressboard effectively removes dirt from oil due to its cellulosic structure, laboratory experiments have demonstrated that its electrical performance is inferior to that of polyurethane.

## Figures and Tables

**Table 1:** Pressboard and Polyurethane Threshold and Peak Values (1 mm).

Material	Voltage (kV)	Peak Value Positive (A)	Peak Count Positive	Threshold Value (A)
Pressboard	14.4	0.44	55	0.42
Polyurethane	14.4	0.46	36	0.44
Pressboard	18	0.54	8	0.52
Polyurethane	18	0.56	8	0.54
Pressboard	21.6	0.68	0	0.68
Polyurethane	21.6	1.04	29	0.68
Pressboard	25.2	2.04	16	0.96
Polyurethane	25.2	1.44	70	1.2

**Table 2:** Pressboard and Polyurethane Threshold and Peak Values (2 mm).

Material	Voltage (kV)	Peak Value Positive (A)	Peak Count Positive	Threshold Value (A)
Pressboard	14.4	0.58	14	0.56
Polyurethane	14.4	0.64	15	0.62
Pressboard	18	0.54	0	0.54
Polyurethane	18	0.54	8	0.52
Pressboard	21.6	0.86	31	0.84
Polyurethane	21.6	1.08	14	0.96
Pressboard	25.2	1.76	40	0.96
Polyurethane	25.2	1.52	92	1.2

**Table 3:** Pressboard and Polyurethane Threshold and Peak Values (3 mm).

Material	Voltage (kV)	Peak Value Positive (A)	Peak Count Positive	Threshold Value (A)
Pressboard	14.4	0.44	7	0.42
Polyurethane	14.4	0.44	3	0.42
Pressboard	18	0.58	89	0.54
Polyurethane	18	0.54	76	0.52
Pressboard	21.6	0.72	25	0.68
Polyurethane	21.6	0.68	0	0.68
Pressboard	25.2	0.96	2	0.88
Polyurethane	25.2	0.88	0	0.88

**Table 4:** Harmonic values of leakage current at 1 mm material thickness.

	Harmonic Values (A)							
	Polyurethane				Pressboard			
Frequency	14.4 kV	18 kV	21.6 kV	25.2 kV	14.4 kV	18 kV	21.6 kV	25.2 kV
(Hz)								
50	0.0193	0.0049	0.0484	0.1654	0.0074	0.0040	0.0468	0.0429
100	0.1920	0.2385	0.2874	0.4649	0.1877	0.2324	0.2843	0.4316
150	0.0009	0.0015	0.0013	0.0216	0.0007	0.0010	0.0011	0.0226
200	0.0028	0.0031	0.0046	0.0081	0.0024	0.0026	0.0042	0.0059
250	0.0008	0.0012	0.0021	0.0019	0.0007	0.0011	0.0022	0.0049
300	0.0136	0.0181	0.0221	0.0308	0.0133	0.0175	0.0213	0.0316
350	0.0010	0.0016	0.0014	0.0029	0.0010	0.0012	0.0019	0.0039
400	0.0099	0.0130	0.0125	0.0229	0.0101	0.0116	0.0142	0.0186
450	0.0007	0.0008	0.0019	0.0028	0.0010	0.0008	0.0015	0.0020
500	0.0073	0.0107	0.0131	0.0216	0.0091	0.0107	0.0151	0.0198
550	0.0015	0.0017	0.0014	0.0038	0.0016	0.0011	0.0016	0.0022
600	0.0058	0.0059	0.0085	0.0124	0.0051	0.0058	0.0080	0.0108
650	0.0004	0.0008	0.0016	0.0035	0.0003	0.0011	0.0012	0.0023
700	0.0056	0.0060	0.0062	0.0178	0.0058	0.0051	0.0088	0.0102
750	0.0012	0.0011	0.0009	0.0035	0.0010	0.0008	0.0015	0.0028
800	0.0056	0.0071	0.0089	0.0128	0.0048	0.0060	0.0060	0.0121
850	0.0005	0.0009	0.0007	0.0037	0.0009	0.0007	0.0012	0.0038
900	0.0033	0.0048	0.0071	0.0096	0.0033	0.0044	0.0045	0.0108
950	0.0007	0.0011	0.0020	0.0024	0.0009	0.0010	0.0014	0.0026
1000	0.0014	0.0006	0.0028	0.0033	0.0003	0.0011	0.0019	0.0083

**Table 5:** Harmonic values of leakage current at 2 mm material thickness.

	Harmonic Values (A)							
	Polyurethane				Pressboard			
Frequency	14.4 kV	18 kV	21.6 kV	25.2 kV	14.4 kV	18 kV	21.6 kV	25.2 kV
(Hz)								
50	0.0392	0.0046	0.1137	0.1759	0.0151	0.0040	0.0329	0.0510
100	0.2707	0.2324	0.4049	0.4777	0.2548	0.2338	0.3791	0.4364
150	0.0038	0.0015	0.0125	0.0205	0.0043	0.0012	0.0182	0.0285
200	0.0034	0.0030	0.0069	0.0072	0.0015	0.0030	0.0057	0.0071
250	0.0018	0.0005	0.0034	0.0018	0.0017	0.0014	0.0048	0.0060
300	0.0188	0.0172	0.0262	0.0309	0.0198	0.0170	0.0286	0.0344
350	0.0026	0.0014	0.0042	0.0040	0.0035	0.0012	0.0046	0.0064
400	0.0123	0.0122	0.0193	0.0214	0.0112	0.0141	0.0160	0.0164
450	0.0016	0.0008	0.0018	0.0024	0.0005	0.0012	0.0035	0.0026
500	0.0125	0.0113	0.0168	0.0211	0.0106	0.0114	0.0149	0.0164
550	0.0032	0.0011	0.0048	0.0030	0.0021	0.0016	0.0028	0.0033
600	0.0062	0.0045	0.0099	0.0122	0.0059	0.0092	0.0057	0.0081
650	0.0019	0.0011	0.0018	0.0029	0.0003	0.0014	0.0011	0.0019
700	0.0085	0.0061	0.0095	0.0147	0.0066	0.0086	0.0084	0.0090
750	0.0024	0.0010	0.0024	0.0029	0.0008	0.0018	0.0014	0.0023
800	0.0071	0.0063	0.0108	0.0140	0.0068	0.0056	0.0089	0.0092
850	0.0015	0.0011	0.0032	0.0035	0.0019	0.0009	0.0024	0.0021
900	0.0064	0.0053	0.0107	0.0137	0.0068	0.0029	0.0113	0.0135
950	0.0012	0.0013	0.0017	0.0030	0.0008	0.0007	0.0021	0.0037
1000	0.0020	0.0003	0.0035	0.0034	0.0042	0.0007	0.0063	0.0051

**Table 6:** Harmonic values of leakage current at 3 mm material thickness.

	Harmonic Values (A)							
	Polyurethane				Pressboard			
Frequency	14.4 kV	18 kV	21.6 kV	25.2 kV	14.4 kV	18 kV	21.6 kV	25.2 kV
(Hz)								
50	0.1876	0.2322	0.281	0.3301	0.1871	0.233	0.2844	0.3304
100	0.001	0.001	0.0017	0.0025	0.0008	0.0009	0.0009	0.0022
150	0.0026	0.0031	0.005	0.0064	0.0023	0.003	0.0045	0.0072
200	0.0011	0.0008	0.002	0.0011	0.0011	0.0012	0.0021	0.0014
250	0.0139	0.0157	0.0214	0.0243	0.0132	0.0179	0.0218	0.023
300	0.001	0.0016	0.0014	0.0016	0.0009	0.0012	0.0014	0.002
350	0.0096	0.0114	0.0134	0.0153	0.0109	0.0136	0.016	0.0204
400	0.0008	0.0008	0.0016	0.0016	0.0008	0.0011	0.0015	0.0019
450	0.008	0.0113	0.0142	0.0154	0.0096	0.0118	0.0156	0.0161
500	0.0016	0.0014	0.0016	0.0011	0.0014	0.0013	0.0016	0.0025
550	0.0066	0.0087	0.008	0.0089	0.0067	0.0082	0.0093	0.0121
600	0.0008	0.001	0.0011	0.0013	0.0006	0.0014	0.0011	0.0008
650	0.0064	0.0036	0.0096	0.0106	0.0077	0.009	0.0114	0.0119
700	0.0012	0.0009	0.0025	0.0009	0.0013	0.0018	0.002	0.0012
750	0.005	0.0089	0.0063	0.0078	0.0037	0.005	0.0071	0.0099
800	0.0005	0.0014	0.0007	0.001	0.0005	0.0013	0.0017	0.0017
850	0.0033	0.0056	0.0046	0.0047	0.0021	0.0028	0.0036	0.0045
900	0.0005	0.001	0.001	0.0009	0.0005	0.0006	0.0012	0.0009
950	0.0013	0.0013	0.0024	0.0026	0.0009	0.0012	0.0007	0.0022
1000	0.0008	0.0004	0.0009	0.0018	0.0008	0.0008	0.0011	0.0008

## Data Availability

All data generated or analyzed during this study can be shared by the corresponding author when there is a demand from researchers.
